# Targeting Glyoxalase-1 Pathway with Natural Compounds: A Translational Strategy to Reduce Dicarbonyl Stress and Prevent Chronic Diseases

**DOI:** 10.3390/life16050822

**Published:** 2026-05-15

**Authors:** Masood Alam Khan, Hina Younus

**Affiliations:** 1 Department of Basic Health Sciences, College of Applied Medical Sciences, Qassim University, Buraydah 51412, Saudi Arabia; 2 Interdisciplinary Biotechnology Unit, Faculty of Life Sciences, Aligarh Muslim University, Aligarh 202002, India; hinayounus@rediffmail.com

**Keywords:** glyoxalase 1, methylglyoxal, oxidative stress, natural compounds, advanced glycation end-products (AGEs), COVID, cancer therapeutics

## Abstract

Methylglyoxal (MG) is a reactive dicarbonyl compound generated mainly as a byproduct of glycolysis. Excess accumulation of MG can promote protein glycation and the formation of advanced glycation end-products (AGEs), which have been associated with oxidative stress, inflammation, mitochondrial dysfunction, and cellular damage. These processes are implicated in the development of several chronic conditions, including diabetes, neurodegenerative disorders, cardiovascular disease, and age-related decline. The glyoxalase system, comprising Glyoxalase I (Glo1) and Glyoxalase II (Glo2), serves as a key cellular defense mechanism that detoxifies MG and helps maintain dicarbonyl homeostasis. Among these enzymes, Glo1 catalyzes the conversion of MG into less reactive intermediates in a glutathione (GSH)-dependent manner. A range of natural compounds and dietary phytochemicals, including sulforaphane, resveratrol, α-lipoic acid, selenium, vitamin D3, and N-acetylcysteine, have been reported to modulate Glo1 activity through transcriptional regulation, antioxidant effects, or support of intracellular GSH levels. Evidence from preclinical and limited human studies suggests that these compounds may help reduce MG burden and AGE formation, although their effects are often indirect and context-dependent. However, several challenges remain, including variable bioavailability, dose-dependent responses, disease-specific differences in Glo1 regulation, and the lack of standardized biomarkers and adequate clinical validation. This review examines the MG–Glo1 axis as a mechanistic framework linking metabolic stress to disease and evaluates natural compounds as context-dependent modulators of this pathway. By integrating mechanistic insights with emerging in vivo and clinical evidence, this work highlights the potential, while acknowledging the limitations, of targeting Glo1 as a translational strategy for managing glycation-associated disorders.

## 1. Introduction

**Figure 1:**
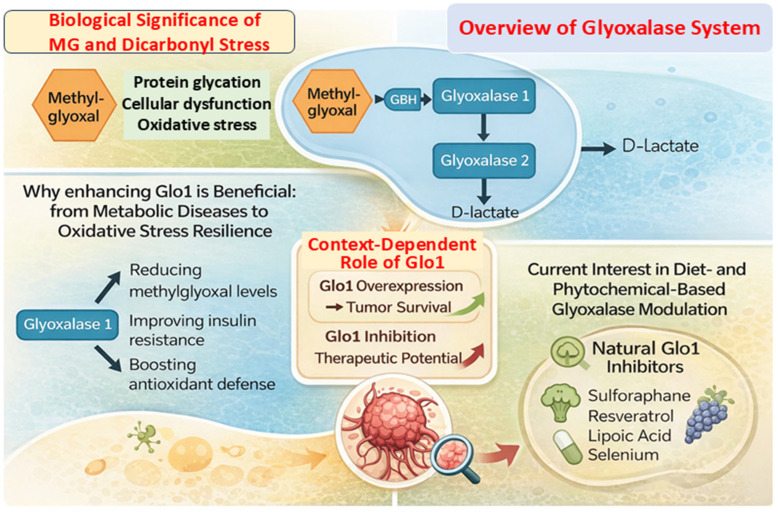
Integrated overview of methylglyoxal (MG) metabolism and the glyoxalase system in health and cancer. MG, a reactive byproduct of glycolysis, can promote protein glycation, oxidative stress, and cellular dysfunction, contributing to dicarbonyl stress. The glyoxalase system, comprising Glyoxalase 1 (Glo1) and Glyoxalase 2 (Glo2), detoxifies MG in a glutathione (GSH)-dependent pathway to form D-lactate. Regulation of Glo1 activity has been associated with reduced MG burden and improved cellular redox balance in metabolic and degenerative conditions. Natural compounds, including sulforaphane, resveratrol, α-lipoic acid, and selenium, may modulate this pathway through direct or indirect mechanisms. In cancer, Glo1 exhibits a context-dependent role: its increased expression in some tumors has been associated with enhanced MG detoxification and cellular survival, whereas its inhibition can elevate MG to cytotoxic levels in experimental settings, suggesting potential therapeutic relevance. This figure is an original schematic representation created by the authors and is not adapted from previously published work.

Methylglyoxal (MG) is primarily formed through the spontaneous degradation of glycolytic triose phosphate intermediates, including dihydroxyacetone phosphate (DHAP) and glyceraldehyde-3-phosphate (G3P): DHAP/G3P → MG + Pi [1]. Although generated at low levels under physiological conditions, the strong electrophilic nature of MG renders it highly reactive. MG readily modifies proteins, lipids, and nucleic acids via non-enzymatic glycation, leading to the formation of advanced glycation end-products (AGEs) that impair molecular structure and cellular function [2]. Excess accumulation of MG and related dicarbonyls contributes to dicarbonyl stress, a pathological state linked to oxidative damage, inflammation, and metabolic dysfunction (Figure 1) [3,4]. MG has been implicated in processes such as insulin resistance, vascular injury, neurodegeneration, and cellular senescence, highlighting its central role in disease-associated metabolic imbalance [4].

Cells counteract MG toxicity through the glyoxalase system, a conserved detoxification pathway comprising Glyoxalase I (Glo1) and Glyoxalase II (Glo2) [5,6]. In this pathway, MG reacts with reduced glutathione (GSH) to form a hemithioacetal intermediate, which is subsequently converted by Glo1 into S-D-lactoylglutathione, representing the rate-limiting step of MG detoxification [7]. Glo2 then hydrolyzes this intermediate to D-lactate while regenerating GSH, thereby maintaining an efficient cyclic detoxification process (Figure 1). Disruption of this system, particularly reduced Glo1 activity, may lead to MG accumulation, enhanced protein glycation, and increased oxidative stress, ultimately compromising cellular viability [8].

Dysregulation of the MG–Glo1 axis has been associated with multiple chronic conditions. In metabolic disorders such as type 2 diabetes, hyperglycemia accelerates MG production, overwhelming detoxification pathways and promoting glycation of proteins involved in insulin signaling [9]. Experimental studies suggest that increased Glo1 expression is associated with improved glucose metabolism and reduced vascular and renal complications [10,11]. Similarly, elevated MG levels have been implicated in neurodegenerative diseases, including Alzheimer’s and Parkinson’s disease, where glycation contributes to protein misfolding, mitochondrial dysfunction, and neuronal damage [12,13]. MG accumulation is also linked to aging-related structural changes in collagen and crystallins, contributing to tissue stiffness and functional decline [14,15]. In vascular systems, MG-induced glycation has been reported to impair endothelial function, reduce nitric oxide bioavailability, and promote atherosclerosis and hypertension [16].

Interest has also focused on the ability of natural compounds to influence the glyoxalase system. Diet-derived phytochemicals have been shown to modulate cellular detoxification processes either by regulating Glo1 expression or by supporting cofactors such as glutathione [8,17]. For example, sulforaphane activates Nrf2 signaling and enhances the expression of antioxidant and detoxification genes, including Glo1 (Figure 1) [18]. Other compounds, such as resveratrol and selenium, have been associated with improved cellular redox balance and maintenance of glutathione levels [19,20]. At the same time, N-acetylcysteine (NAC) and lipoic acid can contribute to intracellular GSH homeostasis [21,22]. In contrast, certain phytochemicals, including curcumin, quercetin, and epigallocatechin gallate, have been reported to inhibit Glo1 under specific conditions [23], indicating that modulation of this pathway may be context-dependent.

Advances in computational and integrative approaches have facilitated the identification of potential Glo1 modulators. Techniques such as molecular docking, molecular dynamics simulations, and pharmacokinetic modeling may enable efficient screening of natural compounds and provide insights into structure–activity relationships, supporting experimental investigation of glyoxalase-targeting strategies. Despite extensive research on MG metabolism and the glyoxalase system, its broader biological and translational implications remain incompletely defined. Moving beyond descriptive accounts of glyoxalase biology, this review establishes the MG–Glo1 axis as a possible unifying mechanistic framework and examines the context-dependent modulation of Glo1 by natural compounds. The review aims to synthesize current evidence on Glo1 regulation and its role in dicarbonyl stress across physiological and pathological conditions.

## 2. Literature Search

A structured and systematic literature search was performed to identify relevant studies on methylglyoxal (MG), Glyoxalase 1 (Glo1), and natural modulators of the glyoxalase system in health and disease. Electronic databases, including PubMed, Scopus, Web of Science, and Google Scholar, were searched for articles published between 2000 and March 2026. The final search was conducted in March 2026. The search strategy combined keywords and Boolean operators, including “methylglyoxal,” “glyoxalase 1,” “dicarbonyl stress,” “advanced glycation end-products (AGEs),” “natural compounds,” “polyphenols,” “Nrf2 activation,” and “Glo1 modulation.”

Studies were included if they (i) provided mechanistic insight into MG detoxification or Glo1 regulation; (ii) reported experimental evidence from in vitro or in vivo models; or (iii) presented clinical or epidemiological data related to glyoxalase function or dietary/phytochemical interventions. Both original research articles and relevant review papers were considered to ensure comprehensive coverage and contextual interpretation. Studies were excluded if they (i) were not directly related to the MG–Glo1 axis; (ii) lacked mechanistic or translational relevance; (iii) were duplicate publications; (iv) were not published in English; or (v) had insufficient methodological detail or incomplete data reporting. Conference abstracts, editorials, and studies with unclear experimental design were also excluded unless they provided uniquely relevant insights. Study selection was conducted independently by both authors using a two-step process: title and abstract screening followed by full-text evaluation, with discrepancies resolved by discussion and consensus. The quality of included studies was assessed based on experimental design, sample size, methodological transparency, reproducibility, and relevance to translational outcomes. Greater weight was given to studies providing quantitative data, validated models, and in vivo or clinical evidence, while findings from limited or preliminary models were interpreted with appropriate caution. As this is a narrative review, no original experimental data were generated. All figures are original schematic representations created by the authors based on the synthesis of the published literature and have not been reproduced from previously published sources.

## 3. Glyoxalase I: Structure, Function, and Regulation

### 3.1. Enzyme Structure and Metal Ion Cofactor (Zn^2+^)

Glo1 is a cytosolic metalloenzyme that predominantly exists as a homodimer [24]. Each monomer comprises approximately 184 amino acids and adopts a characteristic α/β barrel fold commonly observed in enzymes involved in small-molecule metabolism. The active enzyme functions as a dimer, with both monomers contributing to the formation of two catalytic sites at the dimer interface. Its enzymatic activity depends on a divalent metal ion, typically zinc (Zn^2+^) in humans and other mammalian species [25]. The zinc ion is coordinated within the active site by conserved residues, including histidines and glutamates, which help to stabilize substrate binding and facilitate the isomerization reaction. In some bacterial and lower eukaryotic species, the enzyme may utilize other divalent metals such as Ni^2+^ or Co^2+^, demonstrating a degree of metal plasticity. However, zinc remains the predominant cofactor in human Glo1. The role of Zn^2+^ is primarily structural and catalytic [26]. It helps to polarize the carbonyl group of the hemithioacetal substrate, lowering the activation energy for its isomerization to S-D-lactoylglutathione. Loss or replacement of the metal ion with non-catalytic metals results in a significant drop in enzymatic efficiency, indicating its essential role in maintaining the proper geometry and reactivity of the active site.

### 3.2. Role of GSH as a Cofactor and Its Importance in Glo1 Activity

Glo1 does not act directly on free MG but rather on a hemithioacetal intermediate formed spontaneously from the reaction between MG and GSH [1]. This dependence on GSH makes the glyoxalase system inherently linked to the cellular redox state and GSH homeostasis. GSH acts as both a substrate and a cofactor, enabling the transformation of MG into a less reactive intermediate [27]. The reaction catalyzed by Glo1 converts the hemithioacetal into S-D-lactoylglutathione, a more stable and less cytotoxic molecule [8,28]. This product is subsequently hydrolyzed by Glo2 to yield D-lactate and regenerate GSH, completing the detoxification cycle.

The intracellular concentration of GSH significantly influences the rate and capacity of MG detoxification [29]. Under oxidative stress or conditions of GSH depletion, such as in aging, inflammation, or diabetes, Glo1 activity may be compromised, leading to the accumulation of reactive dicarbonyls and AGEs [3]. Therefore, maintaining adequate GSH levels is essential for optimal Glo1 function. This interdependence forms the basis for therapeutic strategies that aim to enhance MG detoxification by replenishing cellular GSH or upregulating its synthesis.

### 3.3. Regulation of Glo1: Transcriptional Control and Post-Translational Modifications

The regulation of Glo1 expression and activity occurs at multiple levels, allowing cells to dynamically respond to changes in metabolic flux, oxidative stress, and environmental stimuli. Transcriptionally, Glo1 is modulated by several transcription factors, among which nuclear factor erythroid 2–related factor 2 (Nrf2) is the most prominent [30]. Nrf2 binds to antioxidant response elements (AREs) in the promoter region of detoxification genes, including Glo1, under conditions of oxidative or electrophilic stress [31]. Activation of Nrf2 by compounds such as sulforaphane or resveratrol has been shown to increase Glo1 mRNA and protein levels in several experimental systems, thereby enhancing the cell’s capacity to eliminate MG [17,18,32]. Other transcriptional regulators include specificity protein 1 (Sp1), which is involved in basal expression, and hypoxia-inducible factors (HIFs), which may influence Glo1 expression under low-oxygen conditions [33,34]. In cancer cells, Glo1 upregulation is often associated with increased expression of transcription factors promoting cell survival, which correlates with elevated glycolytic activity and MG production [35].

**Figure 2:**
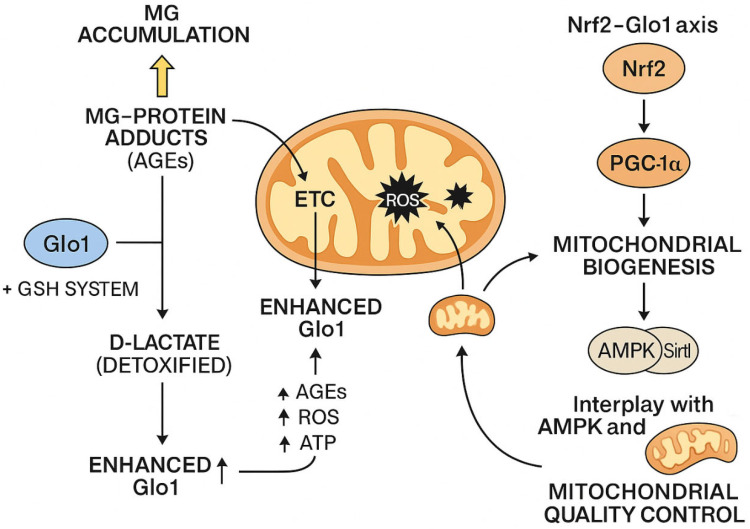
Interplay between MG, Glo1, and mitochondrial function through the Nrf2 signaling axis. Accumulation of MG can promote the formation of AGEs, which are associated with increased oxidative stress, mitochondrial dysfunction, and ROS generation. The glyoxalase system, particularly Glo1, detoxifies MG via a GSH-dependent reaction to form D-lactate, thereby limiting dicarbonyl stress. Enhanced Glo1 activity may reduce AGE and ROS accumulation and help maintain cellular metabolic balance. In parallel, activation of the Nrf2–PGC-1α axis has been associated with improved mitochondrial biogenesis and function, with additional regulation via AMP-activated protein kinase (AMPK) and Sirt1 pathways. These interconnected processes may contribute to mitochondrial quality control and redox homeostasis, although their relative contributions are likely to vary depending on the cellular context. This figure is an original schematic representation created by the authors and is not adapted from previously published work.

Beyond transcriptional regulation, post-translational modifications (PTMs) also modulate Glo1 activity [36,37]. These include phosphorylation, acetylation, and nitrosylation, which may affect enzyme stability, localization, or catalytic efficiency. Phosphorylation of Glo1 by protein kinase A (PKA), for example, can reduce its activity, potentially linking Glo1 regulation to broader signaling pathways. Additionally, epigenetic changes, such as promoter methylation or histone acetylation, have been implicated in tissue-specific or disease-associated alterations in Glo1 expression. These layers of regulation allow for precise control of Glo1 activity in response to both short-term stressors and long-term physiological changes. The interplay between MG detoxification, mitochondrial function, and the Nrf2–Glo1 regulatory axis is illustrated in Figure 2.

### 3.4. Basal and Inducible Glo1 Expression in Various Tissues

Glo1 is ubiquitously expressed across mammalian tissues, reflecting its fundamental role in cellular detoxification [28]. However, expression levels can vary across tissue types, metabolic states, and disease states. High basal expression can be observed in tissues with high metabolic rates and glycolytic activity, such as the liver, kidneys, and brain. These organs are particularly susceptible to MG accumulation and rely heavily on the glyoxalase system for protection. In the central nervous system, Glo1 is expressed in both neurons and glial cells, contributing to neuroprotection against carbonyl stress [37]. In pancreatic islet cells, Glo1 helps maintain insulin secretion and β-cell viability, particularly under hyperglycemic conditions [38]. Muscle tissues, which generate MG during anaerobic glycolysis, have been shown to express Glo1 at significant levels [39].

Glo1 expression is not static and can be induced under various stress conditions. In response to oxidative stress, hyperglycemia, or electrophile exposure, cells upregulate Glo1 via the Nrf2 pathway. This adaptive response appears essential for maintaining proteostasis and preventing cellular damage. Conversely, aging and chronic inflammation, as well as pathological conditions such as diabetes, obesity, neurodegenerative disorders, and cardiovascular diseases, are associated with a decline in Glo1 expression or activity, thereby increasing susceptibility to MG-induced glycation damage. In cancerous tissues, Glo1 is often overexpressed as part of the metabolic reprogramming that supports rapid cell proliferation [40,41]. This overexpression may help tumor cells cope with high MG levels generated through elevated glycolytic flux, thereby enhancing their survival. As a result, Glo1 has been investigated as a potential therapeutic target in oncology, with efforts to selectively inhibit its activity in tumors. In sum, the expression profile of Glo1 reflects both its protective role under physiological conditions and its adaptability under pathological stress. Understanding how its expression is regulated in different tissues and disease contexts provides valuable insight into potential intervention strategies for a range of disorders.

## 4. Mechanisms of Natural Activation of Glo1

**Figure 3:**
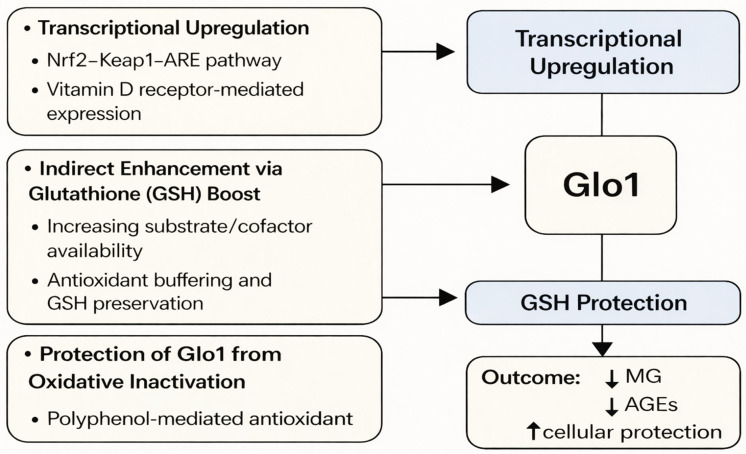
Mechanisms of natural modulation of Glo1. Natural compounds may influence Glo1 activity through multiple complementary mechanisms: (i) direct transcriptional regulation, primarily via the Nrf2–Keap1–ARE pathway and vitamin D receptor (VDR)-mediated gene expression; (ii) indirect enhancement, through increased availability and maintenance of GSH, a critical cofactor required for Glo1 catalytic function; and (iii) protection against oxidative inactivation, whereby antioxidant and polyphenolic compounds may help preserve Glo1 structure and activity. Collectively, these mechanisms may improve the cellular capacity to manage MG-induced dicarbonyl stress. However, the relative contributions of each pathway are likely to vary depending on the compound and biological context. This figure is an original schematic representation created by the authors and is not adapted from previously published work.

The modulation of enzyme systems by natural compounds has emerged as a promising area in molecular nutrition and therapeutic research. Glo1, due to its central role in detoxifying MG and preventing dicarbonyl stress, is one such target for modulation. While most enzymatic studies have historically focused on inhibition, recent findings underscore the potential for natural activation of Glo1 through indirect and direct regulatory mechanisms. These mechanisms are primarily classified into three major categories: transcriptional upregulation of Glo1 gene expression, enhancement of cofactor availability, specifically GSH, and protection of the enzyme from oxidative inactivation through antioxidant support. Together, these processes are proposed to contribute to increasing the functional capacity of Glo1 under physiological and pathological conditions. The major mechanisms underlying natural activation of Glo1 are summarized in Figure 3. These mechanisms are further classified in this review as direct (transcriptional or enzymatic regulation of Glo1) and indirect (modulation via redox balance or glutathione availability).

### 4.1. Transcriptional Upregulation

The regulation of Glo1 at the transcriptional level represents a pivotal mechanism through which natural compounds can increase its expression and, consequently, enzymatic activity [8]. Unlike synthetic enzyme activators, natural bioactives often engage intracellular signaling networks that converge on gene regulatory elements. Two key transcriptional regulators involved in Glo1 gene induction are the Nrf2–Keap1–ARE pathway and VDR-mediated signaling [42].

#### 4.1.1. Nrf2–Keap1–ARE Pathway

Nrf2 is a master transcriptional regulator of antioxidant and phase II detoxification enzymes [43]. Under basal conditions, Nrf2 is sequestered in the cytoplasm by Kelch-like ECH-associated protein 1 (Keap1), which promotes its ubiquitination and subsequent proteasomal degradation [44]. Upon exposure to oxidative stress, electrophiles, or specific natural compounds, modifications to Keap1 cysteine residues result in the release and nuclear translocation of Nrf2. Once in the nucleus, Nrf2 binds to AREs within the promoter regions of target genes, including Glo1 [45]. Several phytochemicals have been shown to activate Nrf2, thereby upregulating Glo1 expression [46]. Sulforaphane, found abundantly in cruciferous vegetables such as broccoli, is among the most well-studied Nrf2 activators [18]. It modifies cysteine residues on Keap1, disrupting the Nrf2–Keap1 complex, and enhances ARE-driven transcription. As a result, Glo1 mRNA and protein levels rise in a range of cell types, including hepatocytes, endothelial cells, and neurons. Importantly, the Nrf2-mediated induction of Glo1 is not merely an adaptive response to sulforaphane’s pro-oxidant effect but a potential cytoprotective response that may enhance the cell’s resistance to glycation stress. Resveratrol, a polyphenol from grapes and berries, and carnosic acid, derived from rosemary, also modulate the Nrf2 pathway, albeit through distinct molecular intermediates, such as the PI3K/Akt or MAPK pathways [47]. Their influence on Glo1 may play a role in neuroinflammation, diabetes, and cardiovascular stress, where Glo1 induction has been associated with improved metabolic resilience and reduced AGE accumulation. While Nrf2 activation offers a powerful route for Glo1 induction, it also necessitates a balance. Prolonged activation may lead to undesired effects such as metabolic reprogramming or immune modulation. Therefore, natural compounds that induce transient, moderate Nrf2 activation are considered favorable for sustained but safe enhancement of Glo1 activity.

#### 4.1.2. Vitamin D Receptor (VDR)-Mediated Expression

Another less explored yet intriguing regulatory pathway for Glo1 expression involves the VDR. The VDR is a nuclear receptor that, upon binding to the active form of vitamin D (1,25-dihydroxyvitamin D3), heterodimerizes with the retinoid X receptor (RXR) and binds to VDREs in the promoter regions of specific genes [48]. Studies have suggested that Glo1 may be a downstream target of VDR activation in certain cell types. In keratinocytes and monocytes, vitamin D treatment has been associated with increased Glo1 mRNA levels, possibly through VDRE elements located in the gene’s promoter or through indirect regulation involving intermediary transcription factors [49]. This upregulation is particularly relevant in inflammatory and immune responses, where vitamin D deficiency is common, and dicarbonyl stress is elevated. Given the widespread deficiency of vitamin D globally and its pleiotropic effects on immunity, metabolism, and oxidative stress, dietary or supplemental vitamin D may serve as a safe and practical strategy for enhancing Glo1 expression, particularly in at-risk populations.

### 4.2. Indirect Enhancement Through GSH Boost

Beyond gene regulation, the availability of cofactors, specifically GSH, is a crucial determinant of Glo1 activity. As GSH is required for the initial conversion of MG into a substrate recognizable by Glo1, its intracellular concentration directly impacts the enzyme’s efficacy. Natural compounds that elevate GSH levels or prevent its depletion can therefore indirectly enhance Glo1 function, even without altering gene or protein expression.

A variety of natural molecules act as precursors or enhancers of GSH biosynthesis. NAC is among the most studied, serving as a cysteine donor for GSH synthesis [50]. While technically a supplement rather than a dietary phytochemical, NAC is derived from the amino acid L-cysteine and is widely found in protein-rich foods. Its administration increases intracellular GSH, thus providing more substrate for the spontaneous formation of the MG–GSH hemithioacetal and supporting Glo1 enzymatic throughput. Other compounds, such as α-lipoic acid, found in spinach and broccoli, can regenerate oxidized GSH (GSSG) back to its reduced form [22]. This recycling helps maintain a high GSH/GSSG ratio, crucial for sustaining antioxidant defenses and enzymatic detoxification. In a randomized trial of 105 diabetic patients, supplementation containing 600 mg α-lipoic acid for three months significantly improved glycemic control, reduced LDL-C and triglycerides, lowered hs-CRP, and enhanced antioxidant defenses [51]. In an animal model of endometriosis, a supplement combination containing α-lipoic acid, NAC, and bromelain reduced inflammatory VCAM1 expression, selectively induced apoptosis in endometriotic endothelial cells, and significantly decreased the number and size of endometriotic cysts, indicating potential therapeutic benefits for inflammatory endometriosis [52]. Additionally, selenium, a micronutrient found in Brazil nuts and seafood, contributes indirectly to Glo1 activity by supporting the synthesis of glutathione peroxidase (GPx), an enzyme that mitigates peroxide accumulation and prevents GSH depletion [53,54]. By lowering oxidative pressure on the GSH pool, selenium helps sustain the cofactor levels necessary for effective Glo1 function.

Many plant-derived antioxidants serve to buffer oxidative stress, thereby preserving GSH levels and ensuring continued Glo1 activity. Natural compounds, such as kaempferol and myricetin, as well as polyphenols, such as chlorogenic acid, scavenge free radicals and ROS, reducing the demand for GSH in non-enzymatic antioxidant reactions [55]. This preservation effect becomes especially important under oxidative load, where GSH is consumed in high amounts for redox regulation. By reducing the burden on the glutathione system, these compounds allow more GSH to remain available for Glo1-mediated detoxification of MG. Thus, even without directly influencing Glo1 expression, dietary antioxidants can significantly modulate the enzyme’s functional capacity.

### 4.3. Protection of Glo1 from Oxidative Inactivation

Enzymes involved in detoxification are particularly susceptible to oxidative modification due to their exposure to reactive species. Glo1, with its reliance on thiol cofactors and metal-ion coordination, can be inactivated by oxidative modifications such as S-nitrosylation, carbonylation, or metal chelation [53]. Maintaining its structural integrity is therefore essential for sustained activity, especially in pathological states marked by chronic oxidative stress. Natural compounds, particularly polyphenols, offer protection against such inactivation through multiple mechanisms. Many polyphenols act as metal ion chelators or radical scavengers, stabilizing both the enzyme and its environment. Epigallocatechin gallate (EGCG) from green tea, for example, can bind free iron and copper ions that would otherwise catalyze Fenton-type reactions, thereby preventing the generation of hydroxyl radicals in the vicinity of Glo1 [19]. Therefore, EGCG may protect Glo1 from oxidative modification without interfering with its functional zinc center. Other polyphenols, such as curcumin and rutin, have been shown to enhance the stability of detoxification enzymes under oxidative conditions [54]. Curcumin, although also studied as a potential Glo1 inhibitor in cancer models, exhibits antioxidant properties that may help stabilize Glo1 in non-cancerous cells. Rutin, a glycosylated form of quercetin, can protect cellular proteins by reducing lipid peroxidation and preserving membrane integrity, thereby preventing secondary oxidative damage to enzymes like Glo1. Additionally, polyphenols often influence cellular redox signaling cascades, activating protective responses via transcription factors such as Nrf2, as mentioned earlier. This dual role, as both indirect gene activators and enzyme stabilizers, makes them particularly attractive as natural modulators of Glo1.

In conclusion, natural activation of Glo1 may result from a complex interplay among transcriptional, metabolic, and protective mechanisms. Compounds that stimulate gene expression via the Nrf2 or VDR pathways may increase Glo1 protein synthesis, while those that support or preserve GSH levels enhance its enzymatic efficiency. Additionally, antioxidants and polyphenols can help in maintaining the structural and functional integrity of Glo1 by preventing oxidative inactivation. Together, these mechanisms offer multiple points of intervention through which dietary and plant-derived compounds can bolster the glyoxalase system. Understanding these pathways not only enhances our appreciation for the protective effects of natural compounds but also guides the rational development of Glo1-targeted strategies in the prevention and management of chronic disease.

## 5. Natural Compounds That Activate or Enhance Glo1

A diverse range of natural compounds derived from foods and medicinal plants has been shown to modulate Glo1 activity [8]. Despite differences in origin, structure, and mechanisms, these bioactives converge in enhancing cellular defense against dicarbonyl stress. This section highlights selected compounds that have emerged as promising candidates for Glo1 modulation, emphasizing their sources, mechanistic relevance, and translational potential (Table 1). Importantly, these compounds are classified as either direct modulators of Glo1 (e.g., via transcriptional or enzymatic regulation) or indirect enhancers that support Glo1 function by modulating cellular redox balance, particularly glutathione availability.

Evidence levels reflect the nature of supporting data, categorized as in vitro (cell-based experiments), in vivo (animal models), and clinical (human studies), with combined categories indicating evidence across multiple levels.

### 5.1. Sulforaphane

Sulforaphane is a naturally occurring isothiocyanate derived from glucoraphanin, a glucosinolate found abundantly in cruciferous vegetables, especially broccoli sprouts [56]. It has attracted extensive research interest due to its broad-spectrum cytoprotective properties and potential role in chemoprevention. One of sulforaphane’s defining features is its rapid absorption and conversion into its active form upon chewing or light cooking, mediated by the enzyme myrosinase. Beyond its general detoxification effects, sulforaphane is widely investigated for its neuroprotective and anti-inflammatory properties, partly because it can cross the blood–brain barrier. Mechanistically, sulforaphane can act as a direct modulator of Glo1 through transcriptional activation, primarily via the Nrf2–ARE pathway. In peripheral blood mononuclear cells (PBMCs), sulforaphane (2.5 µM) has shown a modest transcriptional effect on the glyoxalase system, with a ~1.03-fold increase in Glo1 expression after 48 h, while enzymatic activity remained unchanged. It also induced detoxification responses, with GSTP1 expression increasing by ~1.08-fold at 24 h. Notably, GSH levels decreased significantly, likely due to sulforaphane–GSH conjugate formation, which may transiently influence cofactor availability. These findings indicate that physiologically relevant sulforaphane exposure can induce mild, gene-level modulation of Glo1 rather than effective enzymatic activation [17].

In addition to direct transcriptional effects, sulforaphane may exert indirect effects on the glyoxalase system by modulating cellular redox balance and antioxidant defenses. For example, sulforaphane has been reported to attenuate MG-induced oxidative stress and support glutathione-related pathways, although these effects are not exclusively Glo1-dependent [56]. Thus, its protective actions likely reflect a combination of Glo1-related and broader antioxidant mechanisms. In vivo studies further support its metabolic relevance, showing that sulforaphane (2–10 mg/kg) improves metabolic parameters, including increased insulin levels and HOMA-β, along with reduced fasting glucose and lipid levels, and reduced FGF21, while improving NAFLD and pancreatic integrity [57]. These effects are accompanied by enhanced antioxidant capacity and modulation of gut microbiota. While these outcomes are consistent with reduced dicarbonyl stress, the specific contribution of Glo1 activation remains indirect and requires further validation.

### 5.2. Resveratrol

Resveratrol is a stilbene polyphenol found in red grapes, berries, and peanuts, and is widely recognized for its association with the health benefits of red wine [58]. It gained prominence through the “French paradox,” which links moderate wine consumption to reduced cardiovascular risk despite high-fat diets [59]. Beyond cardioprotection, resveratrol has been reported to influence multiple signaling pathways related to longevity, mitochondrial function, and cellular stress responses, and is often described as a calorie restriction mimetic [60]. In the context of Glo1 modulation, resveratrol exhibits a context-dependent, dual-mode of action. As a direct modulator, trans-resveratrol, particularly in combination with hesperetin (tRES + HESP), has been shown to activate Nrf2 signaling, leading to upregulation of antioxidant response element (ARE)-driven genes that mitigate dicarbonyl stress, oxidative stress, and glycolytic overload. At 5 µM, this combination improved metabolic profiles and reduced markers of inflammation and senescence. Notably, tRES demonstrated ~9-fold greater potency than cis-resveratrol (cRES) in inducing Glo1 activity, suggesting that Nrf2-mediated transcriptional regulation may contribute to enhanced methylglyoxal detoxification [19].

In addition to these direct effects, resveratrol may also exert an indirect modulation of the glyoxalase system through improvement of cellular redox balance and mitochondrial function, which can support GSH availability and overall detoxification capacity. However, these broader antioxidant effects are not exclusively Glo1-dependent and should be interpreted accordingly. In contrast, in MCF-7 breast cancer cells, resveratrol induces cytotoxicity, partly through a ~1.36-fold reduction in Glo1 activity and a ~1.93-fold decrease in mitochondrial membrane potential, indicating mitochondrial dysfunction. Under metabolic stress (antimycin A), it further reduced D-lactate levels (~1.98-fold), reflecting impaired MG detoxification [61]. These findings suggest that, in cancer settings, resveratrol may suppress Glo1 activity, thereby increasing dicarbonyl stress and contributing to cell death. However, these effects are also influenced by broader metabolic and oxidative stress pathways.

### 5.3. Lipoic Acid

Alpha-lipoic acid (ALA) is an organosulfur compound synthesized in small amounts in the human body and obtained from dietary sources such as spinach, broccoli, and organ meats [62]. It serves as a cofactor for mitochondrial dehydrogenase complexes and has been widely used as a therapeutic agent in the management of diabetic neuropathy. Due to its amphipathic nature, ALA can function in both aqueous and lipid environments, enabling broad antioxidant activity and support of cellular redox balance [22]. ALA has also been reported to influence glucose metabolism, making it relevant in metabolic disorders where glyoxalase system imbalance may occur. In the context of Glo1 modulation, ALA primarily acts as an indirect enhancer rather than a direct regulator. Its effects are largely mediated through the restoration and maintenance of intracellular GSH, a critical cofactor required for Glo1 activity. Experimental studies using combination approaches have employed defined concentrations of taurine (10 mM), α-lipoic acid (10 µM), and vitamin B6 (10 µM), which reduce MG-derived glycation and oxidative stress by enhancing antioxidant defenses, particularly GSH availability [63]. This, in turn, supports Glo1-mediated detoxification of MG, thereby protecting oocyte development from dicarbonyl-induced damage. Clinical evidence further supports this indirect role. Supplementation with α-lipoic acid at a dose of 600 mg/day for approximately 3 months has been shown to improve glycemic control and enhance antioxidant status in patients with type 2 diabetes, likely through increased GSH levels rather than direct upregulation of Glo1 expression or activity. These effects may contribute to reduced oxidative and glycation-related stress, although the specific contribution of Glo1 modulation remains indirect and context-dependent [64].

### 5.4. Selenium

Selenium is a trace mineral essential for human health and is naturally found in foods such as Brazil nuts, seafood, eggs, and whole grains [65]. It is incorporated into selenoproteins, including glutathione peroxidases (GPxs) and thioredoxin reductases, which play key roles in maintaining cellular redox balance and detoxifying peroxides. Unlike many plant-derived compounds that act primarily through signaling pathways, selenium functions as an integral component of enzyme systems, contributing structurally to antioxidant defense mechanisms [66]. In the context of Glo1 modulation, selenium acts as an indirect enhancer rather than a direct regulator. Its effects are mediated by maintaining cellular redox homeostasis, particularly by supporting GSH availability and reducing peroxide-induced GSH depletion [67]. By preserving intracellular GSH levels, selenium may facilitate optimal Glo1 activity, as GSH serves as a cofactor for MG detoxification. However, this supportive role is indirect and not specific to Glo1 activation. Epidemiological studies further indicate that selenium status is inversely associated with inflammation, immune dysregulation, and insulin resistance [68]. While these observations are consistent with reduced oxidative and metabolic stress, the contribution of Glo1 modulation in these effects remains associative and requires further mechanistic validation.

### 5.5. Vitamin D3

Vitamin D3 (cholecalciferol) is synthesized in the skin upon exposure to ultraviolet B (UVB) radiation and is also obtained from dietary sources such as fatty fish, cod liver oil, and fortified dairy products [69]. It is metabolized in the liver and kidneys to its active hormonal form, 1,25-dihydroxyvitamin D3 (calcitriol), which exerts its biological effects through binding to the vitamin D receptor (VDR) [70]. While its role in calcium homeostasis and bone health is well established, emerging evidence suggests broader involvement in immune regulation, inflammation, and metabolic processes. In the context of Glo1 modulation, vitamin D appears to exert predominantly indirect and context-dependent effects, with limited evidence for direct enzymatic activation. Its primary actions are associated with the attenuation of AGE–RAGE–mediated inflammatory signaling rather than the induction of the glyoxalase system. At the transcriptional level, vitamin D has been reported to modestly influence genes related to the glyoxalase pathway under specific conditions, with GLO1 expression increased by approximately 2.0–2.3-fold in PBMCs [71,72]. However, these findings have not been consistently translated into changes in protein expression or enzymatic activity, suggesting a limited functional impact.

Clinical evidence further supports an indirect role. Supplementation with 100 µg/day for 3 months in patients with type 2 diabetes mellitus significantly improved glycation-related parameters, including reduced RAGE expression (0.72 vs. 0.95 in placebo; *p* = 0.001), decreased serum AGEs and TNF-α, and a marked increase in circulating vitamin D levels (*p* < 0.001). Notably, changes in GLO1 expression were not statistically significant (*p* = 0.06) [72]. Collectively, these findings suggest that vitamin D primarily modulates glycation-associated pathways through anti-inflammatory and metabolic effects, with only modest or indirect influence on Glo1 activity. While vitamin D may contribute to reducing dicarbonyl stress, its role in supporting Glo1-mediated methylglyoxal detoxification remains limited and context-dependent, warranting further investigation in well-controlled human studies.

### 5.6. N-Acetylcysteine (NAC)

NAC is a thiol-containing compound widely used as a mucolytic agent and as an antidote for acetaminophen poisoning [73]. Although not a classical dietary phytochemical, its derivation from the amino acid cysteine and its widespread use as a supplement place it within the broader category of natural health-related compounds. NAC is a well-established precursor of intracellular glutathione (GSH) and plays a central role in redox regulation [74]. In the context of Glo1 modulation, NAC functions as an indirect enhancer rather than a direct regulator. Its primary mechanism involves replenishment of intracellular GSH, a critical cofactor required for Glo1-mediated detoxification of MG. Thus, the effects of NAC on the glyoxalase system are largely mediated through restoration of cellular redox balance rather than direct activation of Glo1.

Preclinical studies support this indirect role. In diabetic ApoE^−^/^−^ mice, 12-week NAC treatment reduced atherosclerotic plaque size, decreased oxidative stress markers such as malondialdehyde, and increased antioxidant enzymes, including SOD1 and GPx1. These changes were accompanied by improved endothelial function, reflected by enhanced p-Akt, p-eNOS, and nitric oxide levels, suggesting involvement of a GSH-dependent mechanism that supports Glo1 activity [21]. Similarly, in acetaminophen-induced hepatotoxicity, Glo1 deficiency was associated with increased oxidative stress, RAGE activation, and hepatocyte necrosis, indicating a protective role of the glyoxalase system. NAC mitigated this injury primarily by restoring GSH and reducing oxidative stress, which may facilitate MG detoxification. The finding that the GSH surrogate (ψ-GSH) attenuates toxicity in the absence of Glo1 implies that oxidative stress is a predominant contributor to injury, whereas the GSH–Glo1 axis plays a supportive, but not sole, protective role [75]. Collectively, these findings suggest that NAC supports the glyoxalase pathway indirectly through GSH replenishment, with its overall protective effects reflecting broader antioxidant and cytoprotective actions. Its clinical safety profile and accessibility further support its relevance in conditions associated with oxidative stress, including metabolic, neurological, and toxic injury models. However, its effects are not specific to Glo1 modulation.

### 5.7. Carnosic Acid

Carnosic acid is a polyphenolic diterpene found in culinary herbs such as rosemary (*Rosmarinus officinalis*) and sage (*Salvia officinalis*) [76]. It is fat-soluble, heat-stable, and exhibits potent antioxidant activity, particularly in lipid-rich environments. In addition to its role as a direct ROS scavenger, carnosic acid has been reported to activate the Nrf2 signaling pathway, thereby influencing the expression of antioxidant and cytoprotective genes [77]. This dual functionality has attracted interest in neuroprotection and cognitive aging, where oxidative damage to lipids and proteins is prominent. Carnosic acid is also often studied alongside carnosol, suggesting that the combined effects of rosemary-derived compounds may contribute to their overall biological activity. In the context of Glo1 modulation, carnosic acid appears to act primarily as an indirect modulator. Its effects are mediated by a reduction in oxidative and dicarbonyl stress rather than by direct regulation of Glo1 expression or activity. Experimental studies have demonstrated strong anti-glycation activity at 400 µg/mL, with >90% inhibition of fluorescent AGE formation across BSA/glucose, BSA/glyoxal, and BSA/methylglyoxal models. It also reduced Nε-(carboxymethyl)lysine (CML) by approximately 75.2% and Nε-(carboxyethyl)lysine (CEL) by 64.2% in the BSA/glucose system, and further decreased CML (~53.9%) and CEL (~24.3%) in reactive carbonyl models [78,79]. Importantly, carnosic acid reduced MG levels and protein carbonylation, indicating attenuation of dicarbonyl stress. These findings suggest that carnosic acid may support the glyoxalase pathway indirectly by limiting MG accumulation and AGE formation. However, these effects are not specific to Glo1 and likely reflect broader antioxidant and carbonyl-scavenging mechanisms. Thus, while carnosic acid contributes to cellular protection against glycation and oxidative damage, its role in Glo1 modulation remains indirect and context-dependent.

## 6. Potential Applications of Glo1 Activation

The modulation of Glo1 activity has attracted increasing scientific interest across multiple disciplines, including endocrinology, neuroscience, gerontology, and oncology [41]. As an enzyme involved in the detoxification of the reactive metabolite MG, Glo1 contributes to cellular homeostasis. The impact of Glo1 modulation appears to be context-dependent, varying with tissue type, disease stage, and metabolic environment, highlighting the need for careful and targeted approaches in its potential therapeutic applications.

### 6.1. In Metabolic Diseases (e.g., Diabetes, Obesity)

One of the most promising and well-characterized applications of Glo1 activation is in the context of metabolic disorders, particularly type 2 diabetes mellitus and obesity [39]. These conditions are often characterized by chronic hyperglycemia, dysregulated glycolysis, and elevated MG production. Elevated MG levels in diabetic individuals have been linked to insulin resistance, pancreatic β-cell dysfunction, and systemic complications involving the kidneys, nerves, and eyes [80]. A critical site of MG action in diabetes is the pancreatic β-cell, which is rendered highly vulnerable by low antioxidant defenses [81]. MG-modified proteins within β-cells can impair insulin biosynthesis, disrupt granule exocytosis, and lead to mitochondrial dysfunction [81]. Studies in diabetic models have shown that Glo1 activation can improve β-cell viability and insulin secretion, suggesting a potential role in preserving endogenous insulin production [82].

Moreover, MG contributes to peripheral insulin resistance by modifying key components of the insulin signaling cascade, including insulin receptor substrate proteins and downstream kinases [83]. These glycation events impair insulin binding and signal transduction, reducing glucose uptake in adipose and muscle tissue. Enhancing Glo1 activity may help mitigate these effects by preventing the formation of MG adducts in target tissues, thus preserving insulin sensitivity. In the setting of obesity, where metabolic flux and inflammation coexist, Glo1 activity often becomes insufficient to keep pace with elevated MG production. Adipose tissue in obese individuals shows signs of glycation stress, which contributes to adipocyte dysfunction, secretion of pro-inflammatory adipokines, and recruitment of macrophages. Targeted activation of Glo1 in adipose tissue may help break this cycle of inflammation and metabolic disturbance, improving insulin responsiveness and adipose tissue health. Furthermore, diabetic complications such as nephropathy, retinopathy, and neuropathy are closely tied to chronic MG accumulation. Animal studies have suggested that increasing Glo1 expression or activity can reduce the formation of AGEs in kidney and nerve tissues, ameliorating functional decline and slowing disease progression. These findings support the therapeutic value of Glo1 activation as both a preventive and disease-modifying approach in metabolic diseases.

### 6.2. In Neurodegenerative Diseases

The brain is particularly susceptible to dicarbonyl stress due to its high metabolic rate, relatively low regenerative capacity, and abundant lipid content, which is prone to oxidative modification. Neurodegenerative disorders, including Alzheimer’s disease (AD) and Parkinson’s disease (PD), exhibit elevated levels of MG and AGEs in both human samples and animal models. The glycation of neuronal proteins, tau hyperphosphorylation, and mitochondrial dysfunction are among the hallmark features exacerbated by MG accumulation [84]. MG can modify proteins critical for synaptic plasticity, neurotransmitter synthesis, and vesicle transport. In Alzheimer’s disease, MG has been implicated in the promotion of amyloid-β aggregation and tau pathology, two key pathological processes driving cognitive decline. Increased MG levels have been observed in the hippocampus of AD patients, suggesting that impaired detoxification of this compound may be a driver of disease progression rather than a consequence.

In Parkinson’s disease, dopaminergic neurons are especially vulnerable to glycation stress [84]. MG-modified α-synuclein exhibits increased aggregation potential, forming toxic oligomers that interfere with vesicle trafficking and mitochondrial respiration [85]. Preclinical studies indicate that upregulation of Glo1 can reduce α-synuclein glycation, improve mitochondrial function, and protect against dopaminergic neuron loss. Consistent with this concept, α-synuclein ablation has been shown to increase methylglyoxal-driven glycation stress, leading to compensatory induction of Glo1. However, the continued accumulation of AGEs suggests that this adaptive response is insufficient to fully restore mitochondrial metabolic homeostasis, thereby contributing to persistent mitochondrial dysfunction and oxidative stress in the brain [86]. Moreover, chronic MG exposure has been shown to impair long-term potentiation (LTP), a fundamental mechanism underlying learning and memory, through glycation-mediated modification of NMDA receptors and calcium channels. Consistent with this, MG-induced neurotoxicity may disrupt mitochondrial redox homeostasis and neurotransmitter metabolism in the brain. Notably, tryptophan-mediated upregulation of Glo1/Glo2 enhances mitochondrial antioxidant capacity. It promotes MG detoxification, thereby reducing oxidative stress, limiting neuroinflammation, and partially restoring mitochondrial function, all of which are associated with depression-like behavior and memory impairment [87]. Enhancing Glo1 activity could help preserve synaptic function, improve cognitive outcomes, and slow neurodegenerative processes. There is also increasing recognition of MG-induced neuroinflammation, characterized by microglial activation and increased expression of pro-inflammatory cytokines. By reducing the MG load, Glo1 activation may help attenuate these inflammatory cascades and potentially contribute to a more favorable environment for neuronal survival. Given the limited efficacy of current therapies for neurodegenerative diseases, Glo1 modulation, particularly through safe, bioavailable natural activators, holds promise as a disease-modifying adjunct that targets a fundamental molecular pathway involved in neuronal injury.

### 6.3. In Anti-Aging and Longevity

The accumulation of molecular damage over time, driven by oxidative stress, glycation, and mitochondrial dysfunction, is a central feature of aging [88]. MG is a significant contributor to the age-related decline in cellular and tissue function, as it readily modifies long-lived structural proteins, including collagen, elastin, and crystallins. MG-mediated glycation of collagen alters extracellular matrix properties. It promotes myofibroblast differentiation and migration through TGF-β–dependent signaling, suggesting that inadequate detoxification of MG, potentially due to insufficient Glo1 activity, facilitates collagen modification and contributes to the progression of fibrotic remodeling in diabetes [89]. One of the key hallmarks of aging associated with MG is the progressive stiffening of connective tissues, including the skin, vasculature, and extracellular matrix. MG promotes the formation of AGEs and crosslinks in structural proteins such as collagen, reducing tissue elasticity and contributing to skin wrinkling, arterial stiffness, and impaired organ compliance. Consistent with this mechanism, MG-induced dicarbonyl stress has been shown to increase arterial stiffness, oxidative stress, and MGH-1 accumulation, key indicators of vascular glycation damage. Importantly, enhancement of endogenous detoxification pathways, likely through the Glo1-mediated MG clearance system, by Gly-Low mitigates these effects, reducing vascular glycation, limiting cellular senescence, and preserving aortic mechanical integrity [90]. Together, these findings highlight the central role of MG accumulation and insufficient detoxification in connective tissue aging and suggest that boosting Glo1 activity may help maintain collagen integrity and vascular elasticity during aging.

In the ocular lens, MG accumulation contributes to protein aggregation and lens opacity, a major factor in age-related cataracts [91]. Glo1 activation in lens epithelial cells has been associated with reduced MG-derived AGE formation and improved maintenance of lens transparency, suggesting its protective role in ocular aging. Consistently, Glo1 overexpression in the diabetic retina enhances MG detoxification, preventing accumulation of MG-derived AGEs such as CEL and MG-H1, preserving Müller glial and vascular integrity, and thereby reducing retinal microvascular damage and acellular capillary formation associated with diabetic eye pathology [92]. Furthermore, epigenetic aging, often assessed by DNA methylation clocks, is influenced by the accumulation of MG and its effects on DNA and histone modifications. MG can act as an epigenetic modifier by inducing histone H3 methylglyoxalation, thereby increasing chromatin accessibility and promoting gene expression. In contrast, enhanced Glo1-mediated detoxification reduces MG accumulation and histone modification, thereby modulating MG-dependent transcriptional responses and helping maintain epigenetic stability [93]. While research in this area is still developing, interventions that reduce dicarbonyl stress, either through lifestyle or supplementation, may favorably modulate epigenetic aging markers.

In caloric restriction studies, which consistently show lifespan extension, increased Glo1 expression has been noted as part of the adaptive metabolic response [94,95]. This suggests that Glo1 may not only be a downstream target of longevity pathways but also an active mediator of lifespan extension under metabolic stress. The application of Glo1-enhancing interventions in aging populations, particularly through diet or nutraceuticals, may provide a broad-based approach to healthy aging, supporting tissue function, metabolic balance, and resistance to age-related diseases.

### 6.4. In Cancer

**Figure 4:**
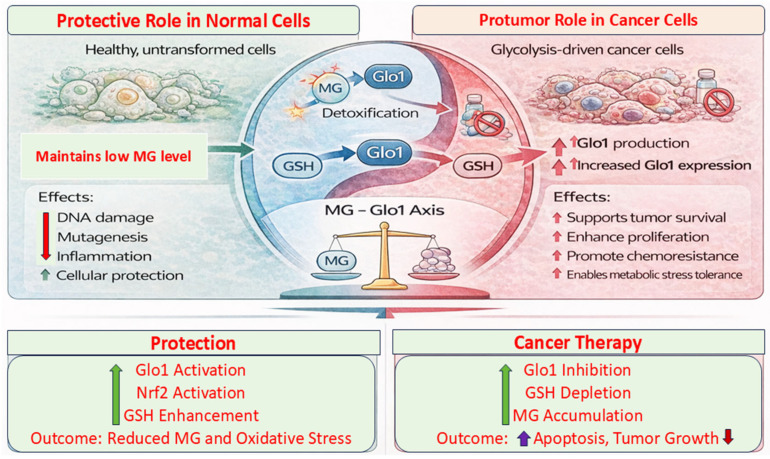
Dual role of Glo1 in cancer and therapeutic targeting. In normal cells, Glo1 detoxifies MG through a GSH-dependent pathway, helping to limit DNA damage, mutagenesis, and inflammatory signaling. In contrast, in cancer cells with elevated glycolytic activity, increased MG production is often accompanied by upregulation of Glo1, which has been associated with tumor cell survival and proliferation. From a therapeutic perspective, Glo1 activation may be protective in normal tissues, whereas its inhibition in tumor cells can increase MG accumulation, leading to cytotoxic stress and apoptosis in experimental models. These context-dependent effects highlight Glo1 as a potential target for selective and tissue-specific modulation in cancer therapy. This figure is an original schematic representation created by the authors and is not adapted from previously published work.

The relationship between Glo1 and cancer is complex and highly context-dependent, positioning it as a context-sensitive regulator in oncology (Figure 4). In normal, non-transformed tissues, Glo1 activation may exert a protective role by detoxifying MG and limiting MG-induced DNA damage, mutagenesis, and pro-inflammatory signaling, thereby potentially reducing the risk of tumor initiation. In contrast, in established cancers, particularly those characterized by high glycolytic flux, elevated MG production is often accompanied by increased Glo1 expression as a metabolic adaptation, which is thought to enable tumor cells to tolerate dicarbonyl stress and support proliferation and survival [41,96]. Conversely, inhibition of Glo1 promotes MG accumulation, which can trigger cellular damage and apoptosis, suggesting that the MG–Glo1 axis may represent a potential biomarker and therapeutic target. However, most supporting evidence remains preclinical [97]. However, most of the current evidence is derived from preclinical and observational studies, and direct clinical validation remains limited.

Importantly, much of the mechanistic evidence for Glo1 function in cancer is derived from diverse tumor types, and its relevance to breast cancer should be interpreted with appropriate caution. While breast cancer models, particularly MCF-7 cells, indicate that modulation of Glo1 can influence mitochondrial function and cell viability, key associations between Glo1 overexpression, enhanced glycolysis, and chemoresistance are primarily based on studies in colorectal cancer, melanoma, and glioma. These observations suggest that Glo1 may act as a metabolic resilience factor in highly glycolytic tumors; however, its tumor-specific roles remain incompletely defined and require further validation across cancer subtypes.

This context-dependent duality presents both opportunities and challenges for therapeutic intervention. In cancer prevention or early-stage disease, maintaining or enhancing Glo1 activity in healthy tissues may help limit glycation-associated genomic instability and inflammatory signaling. In contrast, in advanced or metabolically active tumors, Glo1 overexpression has been associated with a potential survival advantage, likely through more efficient MG detoxification and reduced metabolic stress [41,98,99]. Accordingly, Glo1 inhibition has been explored in experimental settings as a strategy to elevate intracellular MG to cytotoxic levels within tumor cells.

Emerging approaches aim to exploit this dichotomy by selectively modulating Glo1: it is supported in normal tissues but targeted in tumors using context-responsive delivery systems or metabolically guided strategies. However, these approaches remain largely investigational. Glo1 has also been implicated in chemoresistance, as its upregulation may contribute to tumor cell tolerance to therapy-induced metabolic and oxidative stress. In breast cancer, elevated Glo1 expression has been associated with increased glycolytic activity and PKCλ signaling, correlating with tumor progression and poorer clinical outcomes [100]. Conversely, pharmacological inhibition of Glo1 may reduce cancer cell viability and tumor-sphere formation, suggesting a potential role in enhancing chemosensitivity. In addition, modulation of the MG–Glo1 axis may influence the tumor immune microenvironment, as MG may affect antigen processing, MHC expression, and immune cell activation; however, these mechanisms remain incompletely characterized. Overall, current evidence indicates that Glo1 activation may be protective in normal tissues but could also support tumor survival in certain cancers. These findings highlight the need for carefully designed, context-dependent strategies, as well as further validation to define the translational potential of targeting the MG–Glo1 axis in oncology.

## 7. Clinical Repositioning: Glo1 in Post-COVID or Long COVID

**Figure 5:**
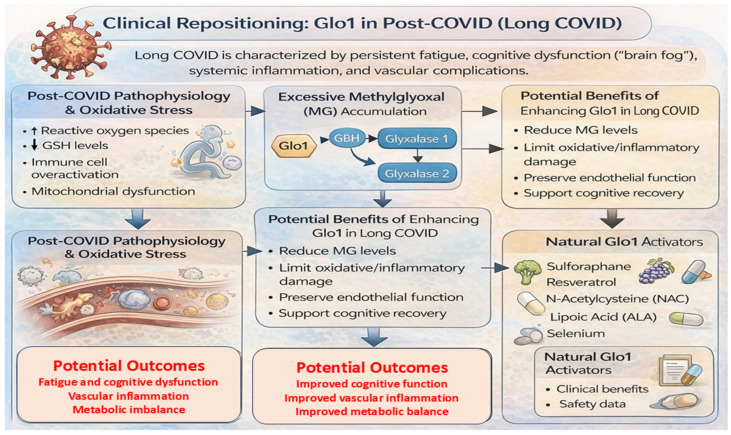
Clinical repositioning of Glo1 in post-COVID (Long COVID). Post-COVID syndrome has been associated with persistent oxidative stress, mitochondrial dysfunction, and immune dysregulation, which may contribute to depletion of GSH and subsequent accumulation of methylglyoxal (MG). Elevated MG is linked to dicarbonyl stress, protein glycation, endothelial dysfunction, and neuroinflammatory responses. Glo1 detoxifies MG in a GSH-dependent way, converting it into less reactive metabolites and thereby contributing to cellular redox balance. Modulation of the Glo1 pathway may help limit MG-associated damage and support vascular and neurological function. Natural compounds with Glo1-modulating and antioxidant properties are being explored as potential adjunct strategies; however, their clinical efficacy in Long COVID remains to be established. This figure is an original schematic representation created by the authors based on the synthesis of the published literature and is not adapted from previously published work.

The global COVID-19 pandemic has not only strained acute care systems but also led to a growing burden of chronic conditions collectively termed post-acute sequelae of SARS-CoV-2 infection (PASC), or Long COVID. Characterized by persistent fatigue, cognitive dysfunction, dyspnea, and systemic inflammation, these symptoms can persist for months after the initial infection [101]. Despite increasing insights into its pathophysiology, effective and targeted therapies remain limited. In this context, modulation of Glo1 has been proposed as a potential and emerging avenue to address post-viral metabolic stress, neuroinflammation, and vascular dysfunction (Figure 5).

A key feature associated with SARS-CoV-2 infection and its sequelae is elevated oxidative stress, driven by mitochondrial dysfunction, cytokine release, and immune dysregulation [102]. This imbalance may lead to GSH depletion, a critical cofactor for Glo1, thereby favoring the accumulation of MG. Increased MG levels have been associated with inflammatory signaling, endothelial dysfunction, and protein glycation, processes that are also reported in Long COVID. Indeed, endothelial abnormalities, including vascular permeability and thrombosis, have been documented in both acute and post-COVID conditions [103]. Given that MG can modify proteins involved in vascular homeostasis, such as endothelial nitric oxide synthase (eNOS) and tight junction components [104,105], it is plausible that enhancing MG detoxification through Glo1 may help support vascular function and limit downstream complications [106]. However, direct evidence in Long COVID populations remains limited.

Neurological manifestations, often described as “brain fog,” represent another prominent feature of Long COVID [107,108]. These symptoms are thought to involve neuroinflammation, blood–brain barrier disruption, and altered neurotransmission. MG has the capacity to cross the blood–brain barrier and modify neuronal proteins, potentially contributing to synaptic dysfunction and inflammatory responses. Evidence from non-COVID models indicates that reduced Glo1 activity is associated with increased MG accumulation and activation of AGE–RAGE signaling pathways. In contrast, restoration of MG detoxification, such as through GSH-enhancing interventions like NAC, can attenuate oxidative and inflammatory damage [109]. While these findings suggest a possible neuroprotective role for the Glo1 pathway, their direct relevance to Long COVID requires further investigation. Similarly, natural compounds with reported Glo1-modulating properties, including resveratrol and sulforaphane, may represent potential candidates, although their efficacy in this context has not yet been established. Chronic fatigue in Long COVID may also be linked to mitochondrial dysfunction and persistent low-grade inflammation, processes that can be influenced by MG accumulation. MG has been shown to impair mitochondrial enzymes and promote AGE formation, which may further amplify inflammatory signaling via receptors such as RAGE [110]. In this framework, Glo1 activation may contribute to restoring metabolic balance, although this remains a hypothesis requiring validation.

From a translational perspective, repositioning natural compounds that influence the GSH–Glo1 axis, including lipoic acid, NAC, selenium, and sulforaphane, offers a conceptually attractive but still exploratory approach. These agents are generally safe and possess antioxidant and anti-inflammatory properties, which may complement their potential effects on dicarbonyl stress. Future studies should focus on evaluating Glo1 activity and MG levels as biomarkers in post-COVID cohorts and determining whether modulation of this pathway translates into measurable clinical benefits. Well-designed clinical trials will be essential to establish causality, optimal dosing, and therapeutic relevance. Overall, targeting the MG–Glo1 axis in Long COVID should be viewed as a promising hypothesis and future research direction, grounded in mechanistic plausibility but currently supported by limited direct clinical evidence. Further investigation is required to determine whether modulation of this pathway can meaningfully contribute to recovery in post-viral syndromes.

## 8. Challenges and Considerations

Despite growing interest in modulating Glo1 for therapeutic purposes, several important challenges must be addressed to translate promising in vitro and in silico findings into safe and effective interventions. These challenges span pharmacokinetic limitations, dose-dependent effects, disease-specific responses, and a lack of clinical validation. Understanding these limitations is crucial for designing future studies, developing functional products, and avoiding unintended consequences of poorly targeted Glo1 modulation.

### 8.1. Bioavailability of Natural Compounds

One of the major challenges limiting the therapeutic application of natural Glo1 activators is their poor bioavailability. Many bioactive compounds identified in experimental studies, such as curcumin, resveratrol, and EGCG, indicate promising activity in cell-based systems but exhibit low oral absorption, rapid metabolism, and short systemic half-lives in vivo. As a result, achieving therapeutically relevant concentrations in target tissues, particularly organs such as the brain or pancreas, remains difficult. For instance, although resveratrol is widely recognized for its biological activity, its systemic availability is significantly limited by extensive first-pass hepatic metabolism, which converts it into glucuronide and sulfate conjugates with reduced biological efficacy. Similarly, curcumin, despite its well-documented antioxidant and anti-inflammatory properties, suffers from poor water solubility and limited intestinal absorption, necessitating relatively high doses to achieve modest plasma levels. To address these pharmacokinetic limitations, several strategies are currently being explored, including the development of advanced delivery systems such as liposomes, nanoparticles, and micelles to enhance stability and gastrointestinal transport, the co-administration of absorption enhancers like piperine to improve intestinal uptake, structural modifications aimed at increasing solubility and metabolic stability, and alternative administration routes such as sublingual or transdermal formulations that bypass hepatic first-pass metabolism. Without such improvements in pharmacokinetic performance, the clinical potential of these compounds to effectively modulate Glo1 activity in humans will remain limited, complicating dose optimization and therapeutic translation.

### 8.2. Dose–Response Relationships and Hormesis

Another important consideration in the therapeutic activation of Glo1 is the non-linear dose–response relationship frequently observed with natural compounds. Many phytochemicals exhibit hormetic effects: their biological effects depend strongly on concentration and exposure duration. At lower doses, these compounds often activate protective cellular stress responses, such as the Nrf2-mediated antioxidant pathway, which can enhance detoxification systems, including Glo1. However, at higher concentrations, the same compounds may exert pro-oxidant or cytotoxic effects, potentially disrupting cellular homeostasis. This biphasic behavior is exemplified by resveratrol, which activates cellular defense mechanisms at moderate concentrations. However, it may induce DNA damage or mitochondrial dysfunction when present at excessive levels. Similarly, sulforaphane stimulates Nrf2 signaling and cytoprotective gene expression at low nanomolar concentrations but can inhibit cell proliferation or trigger apoptosis at higher doses. The hormetic nature of these compounds makes dose optimization both essential and challenging, as small variations in concentration may result in either inadequate Glo1 activation or unintended adverse effects. Furthermore, individual variability in metabolism, age, disease status, and gut microbiota composition can significantly influence the pharmacokinetics and biological responses to these compounds. Consequently, future research should focus on defining clear therapeutic windows, systematically examining biphasic dose-dependent effects across different biological models, and avoiding overgeneralization from high-dose in vitro experiments to clinical or real-world human applications.

### 8.3. Context-Specific Effects of Glo1 Activation

While Glo1 activation is generally beneficial in conditions characterized by oxidative stress, aging, and metabolic dysfunction, its effects are not universally protective. In certain diseases, particularly cancer, elevated Glo1 activity may actually support disease progression. Many tumor cells exhibit increased Glo1 expression as an adaptive response to the high glycolytic flux associated with the Warburg effect, which generates large amounts of MG. By efficiently detoxifying MG, cancer cells can limit glycation-induced protein damage, maintain redox balance, and sustain metabolic activity, thereby promoting tumor growth and resistance to therapy. In such contexts, enhancing Glo1 activity could inadvertently protect malignant cells, increase chemoresistance, or accelerate tumor progression. Consequently, modulation of Glo1 must be context-dependent: activation may be advantageous in aging tissues or neurodegenerative conditions but may not be appropriate in certain cancers. This complexity is further amplified by heterogeneity among tumor types, in which the balance between MG toxicity and Glo1-mediated detoxification varies with tumor metabolic profile, glycolytic dependence, and redox environment. Therefore, therapeutic strategies should avoid indiscriminate Glo1 activation and instead consider disease context, stage, and tissue-specific expression patterns. The integration of biomarkers, such as MG levels or Glo1 activity assays, could guide personalized interventions. Meanwhile, targeted delivery systems may help restrict Glo1 modulation to specific tissues where its activation is therapeutically beneficial.

### 8.4. Need for Clinical Validation and Human Trials

Despite strong preclinical evidence supporting the therapeutic benefits of Glo1 activation, human data remain limited. Most current knowledge is derived from cell culture and animal studies, while well-designed clinical trials evaluating Glo1-targeted interventions in humans are scarce. Although compounds such as sulforaphane and resveratrol have been examined clinically for their antioxidant and anti-inflammatory properties, only a few studies have directly assessed their effects on MG detoxification, Glo1 expression, or AGE-related outcomes. Likewise, observational studies suggesting that higher intake of polyphenols or selenium is associated with reduced disease risk are often influenced by confounding lifestyle and dietary variables. Another major limitation is the lack of standardized biomarkers for assessing Glo1 activity in humans. Currently, there is no universally accepted clinical protocol for measuring Glo1 activity or MG burden, and most available studies rely on indirect indicators such as glycation markers or oxidative stress levels, which may not fully reflect Glo1 function.

Future research should therefore prioritize randomized controlled trials (RCTs) that include Glo1 modulation as a primary or secondary endpoint, alongside the development of validated biomarkers for MG and Glo1 activity in blood, urine, or tissue samples. Long-term evaluation of the safety and efficacy of Glo1-activating compounds is also essential. In addition, exploring synergistic therapeutic strategies, such as combining Glo1 activators with anti-inflammatory agents or mitochondrial-supportive therapies, may enhance clinical outcomes. Without such clinical validation, recommendations for Glo1 activation will remain largely speculative and insufficient for integration into evidence-based medical practice. In conclusion, although activation of Glo1 through natural compounds represents a promising strategy for preventing or managing chronic diseases, several challenges—including improving bioavailability, understanding non-linear dose responses, avoiding inappropriate activation in cancerous tissues, and conducting rigorous human clinical trials—must be addressed before Glo1 modulation can evolve from an experimental concept into a reliable therapeutic approach.

## 9. Future Directions

Future research on Glo1 should prioritize addressing key translational and methodological gaps that currently limit its clinical application. Despite substantial mechanistic understanding, there remains a lack of well-controlled human studies to determine whether modulation of Glo1 translates into meaningful clinical benefit. Carefully designed randomized controlled trials are needed to evaluate the efficacy, safety, and context-specific effects of dietary or pharmacological interventions targeting the glyoxalase pathway. A critical unmet need is the development of validated and standardized biomarkers for assessing MG burden and Glo1 activity in vivo. Reliable quantification of circulating MG, MG-derived AGEs, and tissue-specific Glo1 activity would enable patient stratification, monitoring of therapeutic response, and improved study reproducibility. In parallel, greater emphasis is required on establishing cause–effect relationships, as many current associations between Glo1 modulation and disease outcomes remain indirect.

Future investigations should also focus on defining context-specific roles of Glo1, particularly in conditions where its modulation may have opposing effects, such as cancer versus metabolic diseases. This highlights the need for precision-based approaches that incorporate patient-specific factors, such as age, metabolic status, and disease stage. Advances in targeted delivery systems and tissue-specific modulation strategies may further enhance therapeutic selectivity while minimizing unintended effects. Additionally, systematic evaluation of combination strategies—including agents that influence redox balance, glutathione availability, and transcriptional regulation—may help clarify synergistic or additive effects on the glyoxalase pathway. However, these approaches require rigorous validation to distinguish direct Glo1-dependent mechanisms from broader cytoprotective effects. Overall, integrating mechanistic studies with clinical investigation, biomarker development, and precision medicine frameworks will be essential to determine whether targeting the MG–Glo1 axis can evolve from a conceptual model into a clinically actionable strategy.

## 10. Conclusions

Glo1 plays a central role in cellular defense by detoxifying MG, a reactive metabolite implicated in glycation stress, oxidative damage, and inflammation. Dysregulation of the MG–Glo1 axis has been linked to multiple chronic conditions, including metabolic disorders, neurodegeneration, aging, and cancer, highlighting its importance in maintaining cellular homeostasis. Natural compounds have emerged as promising modulators, supported by preclinical and limited clinical evidence, that influence Glo1 activity through diverse mechanisms, including transcriptional regulation, redox balance, and glutathione homeostasis. Their multimodal actions, combined with favorable safety profiles, make them attractive candidates for modulating dicarbonyl stress in a physiologically compatible manner. However, these effects are often context-dependent, and not all benefits can be attributed solely to direct Glo1 activation. Despite substantial mechanistic evidence, clinical translation remains limited. Key challenges include the lack of well-designed human studies, insufficient biomarker standardization for MG and Glo1 activity, and variability in bioavailability and dosing of natural compounds. Addressing these gaps will be essential for establishing causality and therapeutic relevance. Overall, targeting the MG–Glo1 axis is a promising yet evolving strategy. Future efforts should focus on rigorous clinical validation, biomarker development, and precise delineation of Glo1-specific versus broader cytoprotective effects. Such advances will be critical to determine whether modulation of this pathway can be effectively translated into preventive and therapeutic interventions for glycation-associated diseases.

## Figures and Tables

**Table 1:** Natural Compounds Modulating Glo1: Mechanisms, Mode of Action, and Levels of Evidence.

Compound	Natural Source	Mechanism of Action	Mode of Glo1 Modulation	Level of Evidence	Therapeutic Relevance
Sulforaphane	Broccoli, sprouts, cruciferous vegetables	Activates Nrf2 → binds ARE → increases Glo1 transcription	Direct (transcriptional) + indirect antioxidant	Cell + limited human data	Neuroprotection, anti-inflammatory, oxidative stress resilience
Resveratrol	Red grapes, berries, peanuts	Activates Nrf2 via PI3K/Akt; improves redox balance	Direct (Nrf2-mediated) + context-dependent inhibition (cancer)	Cell + animal + limited human	Cardiometabolic health, anti-aging, glycation control
α-Lipoic Acid (ALA)	Spinach, broccoli, organ meats	Increases intracellular GSH; regenerates antioxidants	Indirect (GSH-dependent)	Animal + human clinical data	Diabetic neuropathy, metabolic disorders
Selenium	Brazil nuts, seafood, cereals	Supports GPx activity → preserves GSH pool	Indirect (GSH maintenance)	Human + epidemiological + animal	Antioxidant defense, insulin sensitivity
Vitamin D3	Sunlight, fatty fish, fortified foods	Activates VDR → binds VDRE → modulates Glo1 expression	Direct (transcriptional, modest) + indirect anti-inflammatory	Human + cell studies	Immune modulation, AGE–RAGE suppression
N-Acetylcysteine (NAC)	Synthetic (cysteine derivative)	Replenishes intracellular GSH	Indirect (cofactor support)	Strong animal + clinical use	Redox therapy, neuroprotection
Carnosic Acid	Rosemary, sage	Activates Nrf2; protects against oxidative inactivation	Indirect + potential direct stabilization	Cell + animal	Neuroprotection, anti-inflammatory

## Data Availability

No new data were created or analyzed in this study.
